# Low glycemic index diet reduces body fat and attenuates inflammatory and metabolic responses in patients with type 2 diabetes

**DOI:** 10.1590/2359-3997000000206

**Published:** 2016-08-31

**Authors:** Júnia Maria Geraldo Gomes, Sabrina Pinheiro Fabrini, Rita de Cássia Gonçalves Alfenas

**Affiliations:** 1 Instituto Federal de Educação, Ciência e Tecnologia do Sudeste de Minas Gerais Barbacena MG Brasil Instituto Federal de Educação, Ciência e Tecnologia do Sudeste de Minas Gerais, Campus Barbacena, Barbacena, MG, Brasil; 2 Centro Universitário de Belo Horizonte Belo Horizonte MG Brasil Centro Universitário de Belo Horizonte, Campus Estoril, Belo Horizonte, MG, Brasil; 3 Departamento de Nutrição e Saúde Universidade Federal de Viçosa Viçosa MG Brasil Departamento de Nutrição e Saúde, Universidade Federal de Viçosa, Viçosa, MG, Brasil

**Keywords:** Glycemic index, inflammation, metabolic profile, diabetes mellitus, body fat

## Abstract

**Objective:**

The aim of this study was to verify the effects of glycemic index (GI) on body composition, and on inflammatory and metabolic markers concentrations in patients with type 2 diabetes.

**Subjects and methods:**

In this randomized controlled parallel trial, twenty subjects (aged 42.4 ± 5.1 years, BMI 29.2 ± 4.8 kg.m^-2^) were allocated to low GI (LGI) (n = 10) or high GI (HGI) (n = 10) groups. Body composition, inflammatory and metabolic markers were assessed at baseline and after 30 days of intervention. Food intake was monitored during the study using three-day food records completed on two non-consecutive weekdays and on a weekend day.

**Results:**

Body fat reduced after the LGI intervention compared with baseline (P = 0.043) and with the HGI group (P = 0.036). Serum fructosamine concentration (P = 0.031) and TNF-α mRNA expression (P = 0.05) increased in the HGI group. Serum non-esterified fatty acids were greater in the HGI than in the LGI group (P = 0.032). IL-6 mRNA expression tended to decrease after the consumption of the LGI diet compared to baseline (P = 0.06).

**Conclusion:**

The LGI diet reduced body fat and prevented the negative metabolic and inflammatory responses induced by the HGI diet.

## INTRODUCTION

The glycemic index (GI) has been used in clinical practice for more than three decades to classify the glycemic impact of foods, meals or diets on glycemic response (1). Its use is supported by the World Health Organization and American Diabetes Association, which recommend the preferential consumption of low GI diets to provide health benefits (1).

Chronic ingestion of low GI diets can prevent and control obesity (2), cardiovascular diseases (3), and type 2 diabetes mellitus (T2DM) (4). By contrast, consumption of high GI diets is related to hyperglycemia and hyperinsulinemia, favoring an increase in glucose uptake by the adipocytes, leading to weight gain and body fat accumulation (5). On the other hand, it has been claimed that daily consumption of two low GI meals can result in beneficial effects on body weight and body composition (6).

High GI diets seem to negatively affect insulin sensitivity and subclinical inflammation, contributing to the pathogenesis of T2DM (7,8). Low GI diets may decrease concentrations of pro-inflammatory biomarkers, especially ultra-sensitive C-reactive protein (CRP), fibrinogen, interleukin-6 (IL-6) and tumor necrosis factor-alpha (TNF-α) (3,7,9). However, there is no consensus among authors regarding these effects (10).

Some studies that evaluated the effect of GI on inflammatory markers are epidemiological (2,11). These studies can detect an association between the variables of interest, but are unable to prove causation (2,11). By contrast, the main limitation of the many clinical trials published is the different macronutrients and dietary fiber contents of the test meals (7). The consumption of diets differing in protein and fat content can lead to different glycemic responses (12). Dietary fiber may also reduce the glycemic response, increasing glucose tolerance (13). Thus, the test meals in such studies must contain the same quantity of fiber and macronutrients so that the observed effect can be attributed to the GI. Due to the lack of consensus in the results of previous studies, we evaluated the effect of the consumption of high or low GI diets for 30 consecutive days on anthropometric, body composition, food intake, glycemic and lipid control, inflammatory marker in concentrations in patients with type 2 diabetes.

## SUBJECTS AND METHODS

### Subjects

**Figure 1:**
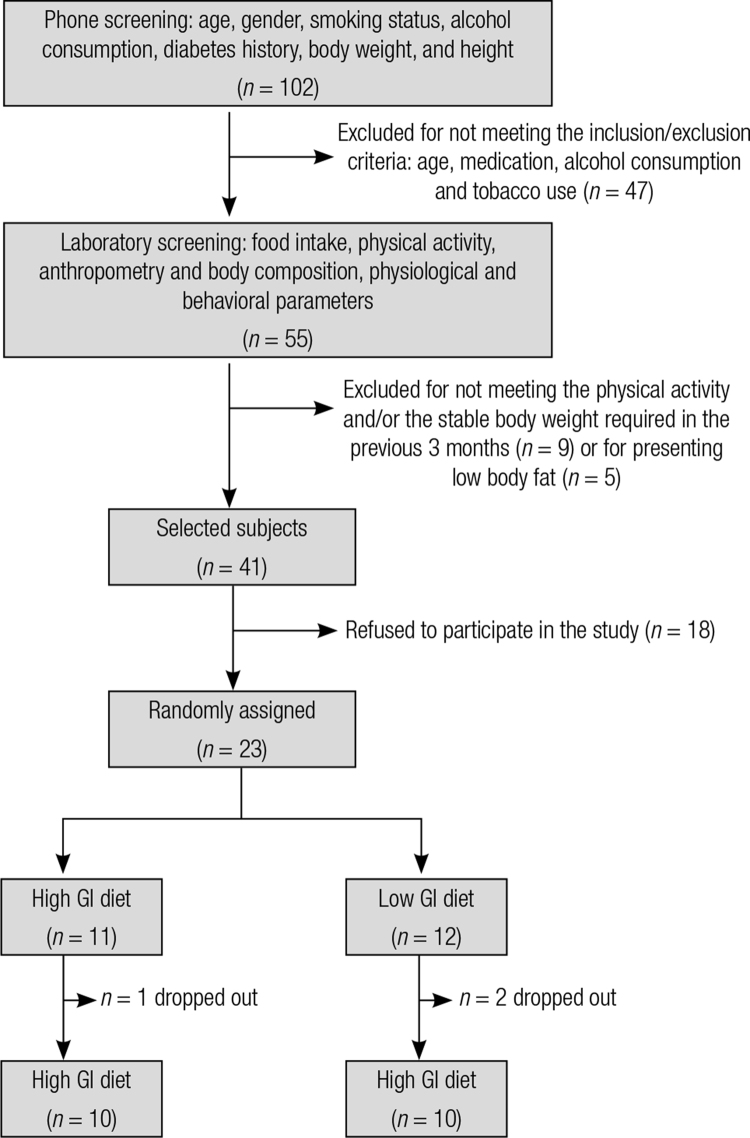
Screening fluxogram.

Subjects were recruited via advertisements in local newspapers, in the university website and flyers distributed around the city of Viçosa, Minas Gerais, Brazil. Data collection took place between April and September 2007. An initial screening was conducted by phone calls and then in the laboratory. Eligible participants were men or premenopausal women between 18 and 55 years old with type 2 diabetes who were receiving biguanides therapy (metformin), which did not change their medications in the previous three months and who had body fat percentage values higher than 16% for men and 24% for women. The exclusion criteria were tobacco use, consumption of > 50 g/day of alcohol (14), pregnancy or lactation, menopause or postmenopause, regular use of hormones, anti-inflammatory medications or other medications that might interfere with outcome measures, recent change (in the previous three months) in the level of physical activity (15) or diet, weight instability (> 3 kg in the previous three months), on a therapeutic diet, dietary allergies or intolerances, cancer, or cardiovascular, renal, or liver disease. Of 102 individuals interested to participate in the study, 55 met the inclusion criteria in the initial screening (via telephone). However, only 41 fully met all the inclusion criteria after screening in the laboratory. Among these, 18 refused to participate due to unavailability to attend daily twice a day to the laboratory during the study. Therefore, 23 subjects were included in the study, and 20 completed the study (Figure 1). This study had a statistical power of 80% (16), considering the baseline mean and standard deviation data presented by the subjects that completed the study, a difference of 2% in body fat content (main variable), and an alpha level of 0.05.

This study was conducted according to the guidelines laid down in the Declaration of Helsinki and all procedures involving human subjects were approved by the Federal University of Viçosa Ethics Committee, Viçosa, Minas Gerais, Brazil (UFV 0382007). Written informed consent was obtained from all subjects. The present trial was registered at www.clinicaltrials.gov, as “Effects of Low- or High-glycemic Index Diets on Metabolic and Inflammatory Responses in Diabetics” (ID no. NCT02383784).

### Experimental design

This was a randomized, single blind (only the subjects were blind), parallel-arm clinical trial. During screening, subjects completed a form to provide demographic, health and habitual physical activity level data. Once selected, subjects were submitted to anthropometric, body composition, food intake and biochemical assessments. Next, they were allocated, according to the order of inclusion and based on the ABBA counter balancing design, to either a high GI (HGI) or low GI (LGI) group.

**Figure 2:**
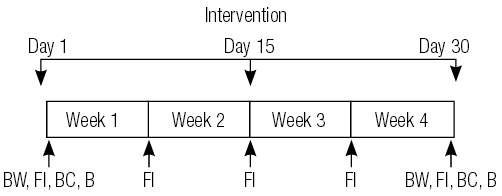
Experimental design. Body weight (BW), food intake (FI), body composition (BC) and biochemical parameters (B) were assessed at baseline and after the experimental period. Food intake was assessed weekly.

Two daily high or low GI test meals (breakfast and an afternoon snack) were consumed in the laboratory during 30 consecutive days. Other meals were consumed in free-living conditions. Subjects received a list discriminating the foods according to their GI values and were instructed to preferentially consume high or low GI foods that corresponded to their experimental group. Food intake was assessed at baseline and weekly throughout the study. Anthropometric, biochemical and body composition parameters were reassessed at the end of the intervention (Figure 2). Subjects were instructed to maintain a constant level of physical activity and to maintain the same type/dose of oral antidiabetic medication during the experimental period.

### Test meals

The test meals’ GIs were determined in a pilot study and it involved 15 healthy subjects (seven men and eight women, mean age of 23 ± 3.2 years, body mass index [BMI] 21.1 ± 2.3 kg/m^2^, nondiabetic, normoglycemic, no family history of diabetes, and not taking medications regularly [except birth control pills]). After 12 hours of overnight fasting, the subjects consumed a portion of the test meals or a glucose solution (reference food) containing 50 g of available carbohydrates within 15 minutes. All subjects consumed the test meals once and the glucose solution was consumed on three different test days by each subject. The test days were separated by a washout period of at least four days (17).

Capillary blood glucose was obtained by a finger-prick at 0 (immediately before meal consumption), 15, 30, 45, 60, 90 and 120 minutes after the start of the consumption of the test meals or glucose solution. The positive area under the glycemic response curve for each test meal was computed by the trapezoidal method and then expressed as a percentage of the average glycemic response of glucose obtained for the same subject. The resulting values were used to calculate the GI of each test meal (12).

The test meals (14 HGI [GI > 70] and 14 of LGI [GI < 55]) (18) had similar energy density, dietary fiber, and macronutrients contents (19) (Table 1). These meals provided 15% of the Estimated Energy Requirements (EER) for each subject (20). The meals’ nutritional compositions were calculated using Diet Pro 5.1i software and based on food label information.

**Table 1:** Mean ± SE test meals glycemic index, available carbohydrate, protein, fat and dietary fiber contents

	Test meals		*P* value

	High GI	Low GI	
GI	74.1 ± 2.9^a^	35.8 ± 3.3^b^	0.010
Energy density (kcal/g)	1.5 ± 0.2	1.5 ± 0.2	1.000
Available carbohydrate (g)	53.7 ± 4.5	53.0 ± 1.1	1.000
Protein (g)	4.9 ± 1.6	4.8 ± 1.5	0.787
Fat (g)	6.4 ± 2.3	6.3 ± 2.3	0.854
Dietary fiber (g)	3.6 ± 1.5	3.0 ± 1.0	0.723

Different letters in the same line indicate statistical difference between groups (*t*-Student test, *P* < 0.05).

Test meals’ GIs (14 types per group) were determined in the laboratory (FAO, 1998). Nutritional composition was obtained using Diet Pro 5.1i Software and food labels. Test meals provided 15% of the Estimated Energy Requirements (EER) for each subject.

GI: glycemic index.

Test meals were composed of a drink, a starchy food, and a fruit. While Corn Flakes^®^ cereal, whole milk, sports drinks, white bread, margarine and papaya were used to prepare the high GI meals, All Bran^®^ cereal, fat free strawberry yogurt, grape juice, multi-grain bread, margarine and apples were the food types used for the low GI versions. Benefiber^®^ (added to high GI meals), glucose (added to HGI meals) and fructose (added to LGI meals) were used to make the test meals nutritionally similar in composition.

### Food intake

Food intake was assessed at baseline and weekly throughout the study, using three-day food records, which were completed on two non-consecutive weekdays and on a weekend day. During the first visit to the laboratory, subjects were instructed on how to complete the food records. Each food record was reviewed with the subjects to ensure data accuracy and completeness. Data was assessed by a single investigator using Diet Pro 5.1i software.

The GI and the glycemic load (GL) of the daily consumed diet (in the laboratory and outside the laboratory) were calculated considering glucose as the reference food (1). For foods not listed in Atkinson and cols. (1), we used GI values of foods presenting similar nutritional composition. Dietary GI and GL were obtained using the following equations (21):



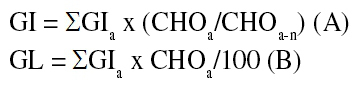



Where GI_a_ represents the GI of a given food; CHO_a_, the available carbohydrate of that same food, and CHO_a-n_, the meal total available carbohydrate content.

### Anthropometry and body composition

Anthropometric and body composition parameters were assessed at baseline and at the end of the intervention. These assessments were conducted by the same investigator, which was trained to ensure precision on data collection. Subjects were asked to wear light clothes, use no accessories, to be barefoot, not to consume water or any other type of food 4 hours before the test, refrain from intense physical activity, avoid caffeine consumption, not use diuretics or drugs that cause water retention in the 24 hours before the test, and not to consume alcohol 48 hours before the test. The assessments were not done in subjects presenting fever, edema or on their menstrual period (women). Upon arriving at the laboratory, participants were instructed to urinate (at least 30 minutes prior to body composition assessment).

Body weight was assessed using a digital electronic scale, with 150 kg capacity and 0.05 kg accuracy (22). Height was determined using an anthropometer fixed to the wall, with 2 m extension and 0.5 cm scale (22). In both procedures, participants stood up barefoot, in erect position, with relaxed arms and head in the horizontal plan. BMI was calculated by dividing body weight (kg) by height squared (m^2^). Waist circumference (WC) was measured with a non-elastic, 2 m extension, 1 mm precision flexible tape measure. WC was assessed in a standing position at the midpoint between the last rib and the iliac crest, and hip was measured at the maximum circumference of the buttocks (23).

Body composition was assessed by tetrapolar bioelectrical impedance (Biodynamics, model 310, TMB). Measurements were taken in the right hemibody, with subjects laid in dorsal decubitus on an isolating surface, without shoes, socks or accessories. The subject’s skin was cleaned with alcohol before placing the electrodes to the hand, wrist, foot and ankle.

### Biochemical assays

Biochemical parameters were assessed at baseline and at the end of the intervention. Serum samples (glucose, total cholesterol, HDL cholesterol, triglycerides, non-esterified free fatty acids (NEFA), ultra-sensitive CRP, insulin, fructosamine, and high molecular weight adiponectin analyses), plasma samples (fibrinogen) and buffy coat (IL-6 and TNF-α) were collected after 12 hours of overnight fasting at baseline and after the experimental period. Samples were centrifuged at 4ºC and stored at -80ºC for later batch analyses.

Glucose, total cholesterol, HDL cholesterol and triglycerides were determined by enzymatic colorimetric tests (autoanalyzer BS200 model, Mindray Bio-Medical Electronics Co., Ltda. Shenzhen, China). Ultra-sensitive CRP was assessed by the immunoturbidimetric method, using the same biochemical analyzer. LDL cholesterol was estimated using Friedewald equation (24).

NEFA were determined by the enzymatic colorimetric method described in the kit Wako^®^ NEFAC (Neuss, Germany). Insulin concentration was measured by the electrochemiluminescence immunoassay (ECLIA) using the Immulite 2000 (DPC^®^) device. High molecular weight adiponectin was evaluated using ELISA kit (EZHMWA-64K, Millipore, Missouri, USES). Fibrinogen analysis was based on Clauss automated method (Fibriquik, brand Organon Teknika). Insulin resistance (IR) was assessed using the HOMA-IR index (Homeostasis Model Assessment-Insulin Resistance) (25).

### Quantitative RT-PCR

Inflammatory markers were assessed at baseline and at the end of the intervention. Analyses of IL-6 and TNF-α were conducted through real-time polymerase chain reaction technique (RT-PCR). Briefly, total RNA (ribonucleic acid) was isolated from buffy coat using TRIZOL reagent (Invitrogen, Paisley, Renfrewshire, UK). High-Capacity cDNA Reverse Transcription Kit (Applied Biosystems, Foster City, California, USA) was used for reverse transcription. Real-time detection of target gene complementary DNA amplification was performed using TaqMan Gene Expression Assays (Applied Biosystems, Foster City, California, USA) for IL-6 (Hs.654458) and TNF-α (Hs.241570). RN18S1 (Hs.03928985_g1) was used as an endogenous reference gene to calculate comparative/delta cycle threshold (DCt) values for IL-6 complementary DNA and TNF-α complementary DNA amplification. The DCt values of target gene amplification were compared with those of an in-house calibrator sample for relative values of gene expression.

### Statistical analysis

Statistical analysis was performed using SPSS software (version 18.0, SPSS Inc., Chicago, IL). The Shapiro-Wilk test (1% significance) was used to evaluate the normality of data distribution. Student’s t-test or the Mann-Whitney U test was used for between groups comparisons, while for paired t-test or Wilcoxon rank sum test was used for within groups comparisons (at baseline and after intervention). Data are presented as mean ± standard deviation (SD) or median (minimum/maximum). The criterion for statistical significance was P < 0.05.

## RESULTS

Twenty patients with type 2 diabetes (10 men and 10 women), aged 42.4 ± 5.1 years old (38 to 49 years old), and mean BMI 29.2 ± 4.8 kg.m^-2^ (22.5 to 37.5 kg.m^-2^) participated in the study. The subjects’ baseline sociodemographic and clinical characteristics are presented in Table 2.

**Table 2:** Baseline sociodemographic and clinical1 characteristics presented by the subjects2

Characteristic	HGI diet (n = 10)	LGI diet (n = 10)	*P* value*
Age (years)	41.1 ± 3.2	44.3 ± 4.8	0.665
Males (%)	5 (50%)	5 (50%)	---
Educational level (years)	8.7 ± 2.5	8.3 ± 2.8	0.723
Disease duration (years)	4.9 ± 1.6	4.8 ± 1.5	0.821
Metformin dosage (mg/day)	655 + 194.2	640 + 171.5	0.671

HGI: high glycemic index; LGI: low glycemic index.

^1^ Other clinical characteristics are presented in Table 3. ^2^ Values expressed as mean ± SD or n (%).

* No statistical difference between groups (student’s *t* test).

The subjects conducted light to moderate physical activity and consumed diets presenting similar macronutrients and dietary fiber contents (50-60% carbohydrate, 15-20% protein, 20-35% fat, and 20-25 g fiber). The diet consumed differed only in term of GI and GL (Table 3). Macronutrient intake was not affected during the study (Table 3).

**Table 3:** Body composition, anthropometry and biochemical outcomes presented by the subjects at baseline and after 30 days of intervention

Outcomes	HGI diet (n = 10)		*P*-value^2^	LGI diet (n = 10)		*P*-value^3^	*P*-value^4^	*P*-value^5^
	
	Baseline	30 days		Baseline	30 days			
Body composition and anthropometry^1^								
				
Body fat (%)	30.1 ± 5.6	29.7 ± 4.3	0.18	33.1 ± 4.9	31.3 ± 4.7	0.043*	0.22	0.036^†^
BMI (kg.m^-2^)	28.6 (25.4/37.5)	28.2 (25.5/36.8)	0.79	28.8 (22.5/33.9)	28.5 (22.5/34.6)	0.83	0.86	0.91
WC (cm)	101 ± 8.7	101 ± 13.4	0.85	99 ± 7.5	98.7 ± 8.5	0.84	0.85	0.61
WHR	0.98 (0.85/1.04)	0.95 (0.86/1.02)	0.89	0.98 (0.86/1.07)	0.97 (0.86/1.04)	0.78	0.97	0.72

**Biochemical parameters^1^**								

Fasting glycemia (mg/dL)	147.8 ± 10.7	157.8 ± 10.4	0.20	148.9 ± 8.2	150.8 ± 8.7	0.36	0.43	0.43
Fructosamine (mmol/L)	1.90 ± 0.05	2.21 ± 0.08	0.031*	1.93 ± 0.04	1.96 ± 0.03	0.23	0.13	0.09
Total cholesterol (mg/dL)	210.1 (180/273.5)	211 (172/284)	0.54	200.4 (123/248.1)	214.1 (145/288.5)	0.15	0.10	0.38
HDL cholesterol (mg/dL)	43 (30/59)	40 (30/54)	0.67	38 (27.6/45.2)	41 (24.5/47)	0.34	0.27	0.76
Triglycerides (mg/dL)	180.2 (88.7/287)	175.3 (132/311.2)	0.09	195 (68/372)	205.1 (63/384.1)	0.09	0.14	0.08
NEFA (mmol/L)	1.0 (0.5/1.5)	1.6 (0.6/1.5)	0.10	1.0 (0.4/1.2)	0.8 (0.6/5.0)	0.22	0.93	0.032^§^
HOMA-IR	4.8 (1.4/10.4)	4.7 (2.1/7.7)	0.87	4.2 (1.2/8.7)	4.3 (1.9/6.2)	0.76	0.34	0.57
Adiponectin (ng/mL)	30.9 (29.8/31.4)	30.8 (30.2/31.6)	0.90	30.1 (29.4/31.3)	30.5 (26.7/93)	0.81	0.78	0.74
Fibrinogen (mg/dL)	289.7 (213.5/333.9)	294.6 (193.6/413.4)	0.35	255.1 (118.5/395.2)	261.3 (141/374.7)	0.48	0.26	0.16
CRP (mg/L)	2.6 (0.8/7.3)	2.8 (0.6/6.13)	0.87	2.7 (0.5/5.5)	2.5 (0.1/6.9)	0.73	0.84	0.44

**Food intake^1^**								

GI	66 ± 4	72 ± 3	0.007*	63 ± 6	54 ± 4	0.005*	0.86	0.001^†^
GL	36.2 ± 10.1	39.3 ± 12.4	0.08	38.6 ± 11.1	32.5 ± 10.6	0.031*	0.75	0.025^†^
Dietary fiber (g)	18.5 ± 5.4	20.6 ± 6.1	0.92	19.6 ± 7.6	21.4 ± 7.2	0.08	0.43	0.53
Carbohydrate (%)	53.5 ± 8.4	57.9 ± 7.7	0.07	59.8 ± 9.3	57.0 ± 8.1	0.33	0.15	0.54
Protein (%)	13.2 ± 1.6	15 ± 2.7	0.09	14 ± 2.0	15.8 ± 2.7	0.67	0.81	0.91
Fat (%)	30.4 + 3.9	34.9 ± 5.9	0.25	31.9 ± 5	34.3 ± 5.2	0.12	0.58	0.83
Energy (kcal/d)	2432.1 ± 581.4	2012.9 ± 591.4	0.08	2217.7 ± 602.4	1997.7 ± 596.2	0.11	0.73	0.85

HGI: high glycemic index; LGI: low glycemic index; BMI: body mass index; WC: waist circumference; WHR: waist-hip ratio; NEFA: non-esterified free fatty acids; CRP: ultra-sensitive C reactive protein; HOMA-IR: Homeostasis Model Assessment – Insulin Resistance; GI: glycemic index; GL: glycemic load. ^1^ Values expressed as mean ± SD or median (minimum/maximum). ^2^ Comparisons between baseline and 30 days after HGI diet. ^3^ Comparisons between baseline and 30 days after LGI diet. ^4^ Comparisons between baseline values (HGI x LGI diet). ^5^ Comparisons between final values (HGI x LGI diet). * *P* < 0.05 (*t*-paired test). ^†^*P* < 0.05 (t test). ^§^*P* < 0.05 (Mann Whitney test).

There were no differences in anthropometric measures, body composition and biochemical parameters between the HGI and LGI groups at baseline. Body fat reduced in the LGI group compared with baseline (P = 0.043) and the HGI group (P = 0.036). LGI group body fat reduced by 1.8% and in the HGI group by 0.4% (Table 3).

**Figure 3:**
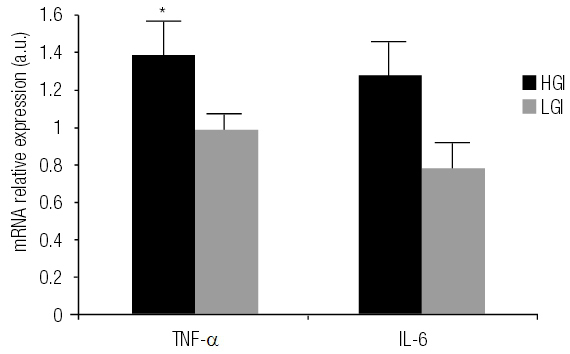
Mean delta ± SD (final – initial values) inflammatory markers expression according to experimental group (n = 10). HGI: high glycemic index diet; LGI: low glycemic index diet; TNF-α: tumor necrosis factor-alpha; IL-6: interleukin-6; a.u.: arbitrary units. * TNF-α mRNA expression increased in the HGI group after intervention (P = 0.05, Wilcoxon test). There is not a significant difference between the changes in TNF-α and Il-6 expression between the groups.

All subjects presented ultra-sensitive CRP concentrations below 10 mg/dL, indicating absence of infection (26). Serum NEFA concentration increased in the HGI group compared to the LGI group after the intervention (P = 0.032). Serum fructosamine concentration (P = 0.031) and TNF-α mRNA expression (P = 0.05) increased in the HGI group at the end of the study. The other biochemical parameters remained unchanged during the study (Table 3, Figure 3).

## DISCUSSION

Consumption of a low GI diet for 30 consecutive days led to greater body fat reduction (1.8%) compared to high GI diet (0.4%). This reduction is desirable, especially among patients with type 2 diabetes, since body fat is positively correlated with cardiovascular disease risk (27). Bouché and cols. (28) also verified a reduction of ~700 g in total fat mass in 11 healthy men after five weeks of LGI. Similar results were observed by Costa and Alfenas (6) in 17 glucose intolerant and excessive body weight subjects in response to 30 consecutive days of LGI hypocaloric diet. In that study, WC decreased after the low GI session (6).

Wee and cols. (29) showed that the consumption of low GI diets favors fat instead of carbohydrate oxidation, leading to body fat reduction. Further, Bouché and cols. (28) observed a reduction on abdominal tissue hormone sensitive lipase (HSL) and on subcutaneous tissue lipoprotein lipase (LPL) gene expression after the consumption of low GI diets. Thus, these authors concluded that the decrease in body fat was not due to increased lipolysis mediated by the HSL, but instead to less fat deposition in the adipose tissue mediated by the LPL (28).

Human LPL promotes plasma triglycerides hydrolysis, increasing circulating NEFA concentrations and its uptake by the adipose tissue. The consumption of high GI diets decreases insulin sensitivity and increases LPL action, since insulinemia is positively correlated with the levels of this enzyme (30). Consequently, although LPL levels were not measured in our study, LPL may have contributed to the increased NEFA concentrations in the HGI group and also to reduce body fat in the LGI group. However, this is only a hypothesized mechanism to try to explain the effects observed in our study.

We verified that HGI diet increased NEFA’s concentrations after the intervention compared to baseline. High concentrations of NEFA appear to inhibit the activity of phosphofructokinase and lead to glucose-6-phosphate accumulation inside the muscle cells, inhibiting cellular glucose uptake (31). The final effect of high serum NEFA concentrations is increased insulin secretion and its reduced action in peripheral tissues, causing beta cells depletion and IR (32). So, the increased serum NEFA and fructosamine concentrations after the intervention may indicate worse glycemic control in the HGI group subjects.

Opperman and cols. (33) assessed the effect of consuming diets differing in GI in a randomized clinical trials meta-analysis. The consumption of low GI diets led to a reduction of fructosamine concentrations compared to high GI diets (33). In our study, although the consumption of the high GI diet increased fructosamine concentrations, the opposite effect did not occur in response to the low GI diet. It is possible that the duration of the present study was not long enough to cause that reduction. Robert and Ismail (34) observed that the GI was useful to evaluate the glycemic response in patients with type 2 diabetes to individual high-carbohydrate foods and to mixed meals (n = 10). However, it must be highlighted that many factors can affect the GI value of foods, such as climate, soil, preparation, cooking time, temperature and acidity (12,13). Therefore, the values obtained in the laboratory under controlled conditions may not be reflected when these same foods are consumed in free living conditions. However, the consumption of two HGI or LGI meals associated with the instruction to preferentially consume foods presenting the same GI of each subject’s study group was sufficient to ensure that diets consumed during our study differed in GI. We also verified elevated TNF-α mRNA expression in the HGI group. TNF-α action may dramatically increase IR and affect glycemic control. TNF-α production is usually increased in obese subjects and its production by adipose tissue is one of the causes of IR (35). This cytokine plays an important regulatory role on adipose tissue fat accumulation (36). TNF-α inhibits LPL action and induces HSL increase, stimulating lipolysis in the adipocytes (36), and consequently increasing circulating NEFA concentrations, as observed in our study. Moreover, TNF-α reduces glucose transporters GLUT 1 and 4 expressions, contributing to IR (35).

Frost and cols. (7) assessed the effect of the GI on insulin sensitivity and TNF-α production in women with a high risk of heart disease. Twenty-eight premenopausal women participated in the study and randomly consumed, for three weeks, isocaloric high or low GI diets presenting similar macronutrients and dietary fiber contents. At the end of the study, there was an increase in insulin sensitivity in response to the consumption of the low GI diet. Adipocyte TNF-α production was higher among people with a family history of cardiovascular disease, but was not affected by GI. However, the GI of the consumed diets was estimated based on food records completed only in the last week of the study, which may not reflect the diet consumed during the study. In that study, the GI was estimated considering the values presented in international tables of GI, instead of being determined in the laboratory, as we did in our study. Consequently, there is no guarantee that the GI values assigned to the test diets were accurate. The GI can be affected by factors such as fruit ripeness, food processing and interactions between nutrients of a mixed meal (37). It has been verified that mixed meals’ GI estimation based on such types of tables may not predict the GI directly measured in the laboratory (38).

The small sample size of our study limited the statistical power to conduct a multivariate statistical analysis. However, the randomization process was carefully conducted by us. Because of that, the intervention groups (HGI and LGI) presented similar baseline body composition, besides clinical, biochemical, and anthropometric data. The wide variance in BMI could also be considered another limitation of our study. Although there was a wide variance in the BMI of our subjects, the baseline values presented by groups was not statistically different. That is, the wide range of variation occurred in both groups. We also emphasize that high body fat percentage (up to 16% for men and 24% for women) instead of BMI was considered as a criterion for inclusion in the study.

In conclusion, while the consumption of a high GI diet for 30 consecutive days caused an increase in fructosamine, NEFA and TNF-α concentrations, consuming a low GI diet caused a significant reduction of approximately 2% in body fat among overweight patients with type 2 diabetes. These results suggest that the consumption of low GI diets can help reduce body fat and prevent the harmful inflammatory and metabolic changes induced by high GI diets.
